# Molecular Cloning and Characterization of Five Glutathione S-Transferase Genes and Promoters from *Micromelalopha troglodyta* (Graeser) (Lepidoptera: Notodontidae) and Their Response to Tannic Acid Stress

**DOI:** 10.3390/insects11060339

**Published:** 2020-06-01

**Authors:** Fang Tang, Huizhen Tu, Qingli Shang, Xiwu Gao, Pei Liang

**Affiliations:** 1 Co-Innovation Center for Sustainable Forestry in Southern China, Nanjing Forestry University, Nanjing 210037, China; tuhuizhen@live.cn; 2 College of Forestry, Nanjing Forestry University, Nanjing 210037, China; 3 School of Agricultural Science, Zhengzhou University, Zhengzhou 450001, China; shangqingli@163.com; 4 Department of Entomology, China Agricultural University, Beijing 100193, China; gaoxiwu@263.net.cn (X.G.); liangcau@cau.edu.cn (P.L.)

**Keywords:** *Micromelalopha troglodyta*, glutathione S-transferases, tannic acid, promoter, transcriptional regulation

## Abstract

Plants accumulate phenolic compounds such as tannic acid to resist insect herbivores. The survival of insects exposed to toxic secondary metabolites depends on the detoxification metabolism mediated by limited groups of glutathione S-transferases (GSTs). *Micromelalopha troglodyta* (Graeser) (Lepidoptera: Notodontidae) is an important foliar pest of poplar trees. GSTs play an important role in xenobiotic detoxification in *M. troglodyta*. Five GST genes were identified in *M. troglodyta* and were classified into five different cytosolic GST classes, delta, omega, sigma, theta, and zeta. Real-time fluorescent quantitative polymerase chain reaction (qPCR) was used to determine the mRNA expression of the five cloned GSTs in the midguts and fat bodies of *M. troglodyta*. The mRNA expression of the five GSTs was significantly induced when *M. troglodyta* was exposed to tannic acid. To further understand the tannic acid regulatory cascade, the 5′-flanking promoter sequences of the five *MtGST*s were isolated by genome walking methods, and the promoters were very active and induced by tannic acid. In summary, the induction of GST mRNA expression was due to the response of five *MtGST* promoters to tannic acid. Therefore, *MtGST* promoters play an important role in the regulation of GST transcription.

## 1. Introduction

The rapid growth and spread of the poplar tree account for its widespread cultivation and naturalization throughout the world. The wood is commonly used as fuel, but is also used for furniture, panelling and plywood production [1]. Poplar trees are vital to the forests of China, and *Micromelalopha troglodyta* (Graeser) (Lepidoptera: Notodontidae) is an important foliar pest of poplar trees. Due to the high fecundity and rapid growth of this insect, infestations usually lead to huge economic losses. Because of many kinds of plant secondary metabolites in poplars [1], the host chemical defence was also the main pressure for these pests in addition to the pesticide stress. Detoxification enzymes, including glutathione S-transferases (GSTs; EC2.5.1.18), are important parts of adaptation mechanisms. GSTs act in physiological roles by initiating the detoxication of potential alkylating agents and interacting with kinases, and they simultaneously bind a wide range of endogenous and exogenous ligands in non-catalytic roles [2,3,4,5]. GSTs are classified as cytosolic, mitochondrial and microsomal GSTs, and insect cytosolic GSTs are further classified into six different classes, including sigma, delta, epsilon, zeta, theta and omega [6,7]. Members of the delta and epsilon classes have been implicated in resistance to pesticides, e.g., organophosphates, organochlorines and pyrethroids [8], while the omega, theta and zeta classes appear to be involved in other cellular processes, including protection against oxidative stress [9].

The presence of plant secondary metabolites in particular plant families may be one factor mediating co-evolution between plants and their related insects [2]. The relationship between plant secondary metabolites and insect GSTs is complex. On the one hand insect GSTs may be involved in detoxifying plant secondary metabolites that are produced during feeding, but on the other, plant secondary plant compounds are also inducers of GSTs in vivo [10,11]. Robert et al. reported that the transcripts and accumulation of proteins of the *DpGSTs* were increased in *Dendroctonus ponderosae* fed with fresh phloem [12,13]; Dai et al. found that the transcripts of *DaGSTs1* and *DaGSTs2* were upregulated after feeding on host phloem in male adults of *Dendroctonus armand* [14]; Huang et al. showed that the expression of GST genes were induced by xenobiotic compounds and bacteria [15].

In gene expression and regulation, the promoter acts as an important *cis*-acting element and is also part of one of the most significant regulatory mechanisms in gene transcription, able to limit the temporal and spatial regulation of gene expression [16]. To understand molecular mechanisms, many promoters have been identified. For example, the *Slgste 1* promoter was found to play an important role in regulating gene expression in response to phytochemicals and insecticides in *Spodoptera litura* [17]; in the promoter region of the *SeGST* gene from *Spodoptera exigua*, *cis*-acting elements responded to chlorpyrifos and cypermethrin [18].

Tannic acid, a plant secondary metabolite in poplars, is a plant polyphenol that is commonly distributed in many plants. As a plant allelochemical, tannic acid causes various types of effects in animals [19]. In vitro tannic acid has an inhibitory effect on GSTs. Zhang and Das reported that plant polyphenols inhibited the rat liver GST [20]; Tang et al. showed that tannic acid inhibited GST activity in two moth species [21]. However, in vivo GSTs in insects have been considered to be responsible for the detoxification of and recovery from oxidative stress agents [22]. The induction of GSTs is a mechanism by which organisms adapt to chemical stress. Chen et al. showed that GST activities were induced by tannic acid in *Helicoverpa armigera* (Hübner) [23]. We reported that GST activities were induced by tannic acid, and cloned the first GST gene in *M. troglodyta, MtGSTd1* [19].

Little is known about GSTs in *M. troglodyta*, therefore, we have chosen to investigate them in our laboratory. The tissue-specific expression of the GSTs of *M. troglodyta* has been studied, and the midguts and fat bodies have higher GST activities than the head and integument [24]. In vivo GST activities were induced by tannic acid in *M. troglodyta* [25]. Furthermore, *MtGSTd1*, the first GST gene identified in *M. troglodyta*, was cloned and characterized in our laboratory [19]. However, there have been no previous reports on the regulatory mechanism of GST expression in *M. troglodyta*. To understand the regulatory mechanism controlling GST expression in *M. troglodyta*, we cloned 5 GST genes and 5 *MtGST* promoters. Then, we assessed the response of mRNA expression of 5 GST genes to tannic acid and measured the response of 5 *MtGST* promoters to tannic acid. We address the following questions in this study: (1) How does tannic acid affect the expression of *MtGST* mRNA and (2) how do the 5 *MtGST* promoters respond to tannic acid? These studies are very important in understanding the regulatory mechanism of *MtGST* mRNA expression in *M. troglodyta*. These data are important for understanding the tannic acid regulatory cascade and for the development of an effective integrated pest management program for *M. troglodyta*.

## 2. Materials and Methods

### 2.1. Insect and Cell Cultures

The larvae used in these assays were from a colony initially established by collecting *M. troglodyta* from poplar trees in Nanjing (31°56′17.00″ N, 118°22′35.98″ E), Jiangsu Province, China. Larvae were supplied fresh poplar leaves in a room maintained at 26 ± 1 °C and 70%–80% relative humidity with a photoperiod of 16:8 (light: dark). The induction of GST mRNA expression by tannic acid was studied using larvae feeding on poplar leaves that had been immersed in tannic acid solutions. Tannic acid (Sigma Chemical, St. Louis, MO, USA) was dissolved in a small aliquot of ethanol and then serially diluted in distilled water to test concentrations of 0.001, 0.01, 0.1, 1, and 10 mg/mL. Freshly collected poplar leaves were immersed in the various solutions for 10 s respectively and then allowed to air-dry. Once dried, two treated leaves were placed into triangular bottles with 5 newly molted third instars, and 10 triangular bottles were set up per concentration, then placed on a shelf in a rearing room. Controls consisted of larvae feeding on leaves immersed in distilled water. Larvae were allowed to feed on treated leaves for 96 h, and then they were collected for dissection. The larvae of *M. troglodyta* were dissected, and the fat bodies and midguts were separated on ice. Firstly, the fat bodies were collected. Secondly, after the peritrophic membrane containing midgut contents were removed, midguts were washed in 1.15% ice-cold KCl and collected. All experiments were independently conducted three times.

Sf9 cells were routinely cultured in SF-900 II serum-free medium (Invitrogen, Carlsbad, CA, USA) supplemented with 10% heat-inactivated fetal bovine serum (HyClone-QB Perbio, Logan, UT, USA), 50 U/mL penicillin, 50 µg/mL streptomycin and 12 µg/mL gentamycin (Invitrogen) at 28 °C.

### 2.2. Cloning the cDNA Sequences of the Glutathione S-Transferases (GSTs)

Total RNA was extracted from one 5th instar larvae using TRIzol reagent (Invitrogen, Thermo Fisher Scientific, Inc., Waltham, MA, USA) according to the manufacturer’s instructions. Total RNA was then treated with DNaseI (TaKaRa, China), and cDNA was synthesized using the PrimeScript 1st Strand cDNA Synthesis Kit (TaKaRa, China). Primers (F and R) were designed and used in polymerase chain reactions (PCR) to amplify the full-length open reading frame (ORF) of GSTs (Table 1). PCR was performed as follows: 94 °C for 5 min; 30 cycles of 94 °C for 30 s, 53 °C for 30 s, and 72 °C for 1 min; and 72 °C for 10 min. The PCR products were purified using the Generay DNA Recovery kit (TaKaRa). The purified cDNA fragments were ligated to pMD19-T vectors (TaKaRa) and cloned into DH5α competent cells. At least three clones were sequenced by the GenScript (Nanjing, China) Biological Technology (Co., Ltd.). For each plasmid insert, both strands of DNA were sequenced at least twice.

### 2.3. Sequence Identity and Phylogenetic Analysis

The amino acid sequences of the GSTs were deduced from their cDNAs and aligned using ClustalX2. Alignments were converted to meg files using MEGA software. A phylogenetic tree was constructed by the neighbour-joining method using MEGA 6.0 [26].

### 2.4. Induction of GST Transcription in Micromelalopha troglodyta by Tannic Acid

Quantitative real-time PCR (qPCR) was performed on a 7500 Real-Time PCR system (Applied Biosystems, Foster, CA) to compare the expression of *MtGST* mRNA in the midguts and fat bodies of *M. troglodyta* larvae exposed to tannic acid and the control. Total RNA for expression analysis was extracted from 50 mg midguts or fat bodies. The integrity and quality of total RNA were tested by running 1% agarose gel electrophoresis and measuring absorbance at 260 and 280 nm using a Thermo Scientific NanoDrop2000. According to the instructions of PrimeScript RT reagent Kit with gDNA Eraser, 5μg total RNA was used to reverse-transcribe into cDNA and then stored at −80 °C or used for the determination of GST mRNA expression. *MtGST* mRNA expression was studied using the SYBR^®^ Premix Ex Taq^TM^ II (Tli RNaseH Plus) kit (TaKaRa Biotechnology (Dalian) Co., Ltd.). Primers (Q-F and Q-R) were designed based on the GST sequences (Table 1). The specificity and sensitivity of these primers were evaluated through melting curve analysis, and the amplification efficiencies were calculated from the standard curves. The amplification efficiency of each pair of primer used in qPCR was within the range of 90%–105%. The amplification of cDNA by qPCR was performed in a 20 μL mixture that contained approximately 1 μL of cDNA, 10 μL of SYBR Premix Ex Taq, 0.4 μL of Rox reference dye (503), 0.4 μL of both sense primer (10 μM) and antisense primer (10 μM) of GST, and 7.8 μL of double-distilled water. *Actin* was used as an internal standard (0.4 μL for each). The qPCR conditions were as follows: 95 °C for 30 s followed by 40 cycles of 95 °C for 5 s and 60 °C for 34 s. To confirm the amplification of specific products, melting-curve cycles were performed with the following parameters: 95 °C for 15 s, 60 °C for 1 min, and 95 °C for 15 s. All experiments were independently conducted four times. The transcript levels of the target genes were expressed as normalized transcript abundances using actin as the internal reference gene. Relative gene expression was calculated using the 2^−ΔΔCt^ method [27].

### 2.5. Cloning the Sequences of the Five GST Promoter Genes

Genomic DNA was extracted from individual larvae using DNA isolation reagent (TaKaRa, China). The promoters were cloned using the Genome Walker^TM^ Universal kit (Clontech, Mountain View, CA, USA). Primers (1 and 2) were designed according to the manufacturer’s instructions (Table 1). A two-step PCR was performed as follows: 94 °C for 5 min; 7 cycles of 94 °C for 25 s and 72 °C for 3 min; 32 cycles of 94 °C for 25 s and 67 °C for 3 min; 67 °C for 7 min; 94 °C for 5 min; 5 cycles of 94 °C for 25 s and 72 °C for 3 min; 30 cycles of 94 °C for 25 s and 67 °C for 3 min; followed by 67 °C for 7 min.

The PCR products were cloned into the pMD-19T vector (TaKaRa, China), transformed into *DH5a* competent cells and sequenced by the Nanjing GenScript Biotechnology Company. The searches for homologous sequences were performed using BLASTN against the National Center for Biotechnology Information database (http://www.ncbi.nlm.nih.gov/).

### 2.6. Analysing the Sequences of the Five GST Promoter Genes

The promoter predictions for the sequences with a score cut-off of 0.80 were conducted with the Berkeley Drosophila Genome Project (BDGP) database (http://www.fruitfly.org/seq_tools/promoter.html). The transcription factor binding sites were predicted by constructing matrices on the fly from TRANSFAC 4.0 sites using TRANSFAC 4.0 software on the AliBaba 2.1 database (http:// www.gene-regulation.com/pub/programs/alibaba2/index. html) with specific parameters (Pairsim to know sites value is 64, match width in bp value is 10, minimum number of sites is 5, minimum match conservation value is 75%, similarity of sequence to match value is 100%, and factor class level is 4).

### 2.7. Construction of the MtGSTd2 Promoter-PGL 4.10, MtGST01 Promoter-PGL 4.10, MtGSTs1 Promoter-PGL 4.10, MtGSTt1 Promoter-PGL 4.10 and MtGSTz1 Promoter-PGL 4.10 Constructs

To generate a construct fusing the *MtGST* promoters with the luciferase reporter gene *luc2* (*Photinus pyralis*), primers (F-1 and R-1) with Xho I and Nhe I sites were designed based on the promoter sequence (Table 1). The PCR products and reporter genes were digested by Xho I and Nhe I digestion. Thus, five recombinant plasmids, *MtGSTd2* promoter-PGL 4.10, *MtGSTo1* promoter-PGL 4.10, *MtGSTs1* promoter-PGL 4.10, *MtGSTt1* promoter-PGL 4.10 and *MtGSTz1* promoter-PGL 4.10, were obtained.

### 2.8. Transient Transfection and Dual Luciferase Assay

Sf9 cells were seeded onto a 96-well plate (9 × 10^4^ cells/well) and transiently cotransfected with *MtGST* promoter-*PGL 4.10* luciferase reporter constructs (200 ng/well) and the internal *renilla* luciferase reporter plasmid phRL-TK (Promega; 20 ng/well) using Cellfectin II reagent (Invitrogen; 1 μL/well). The transfection efficiency and luciferase activity were determined as described by Peng et al. [28]. After 48 h, the cells were harvested, and the resulting lysates were used to measure the renilla and firefly luciferase activities with a Dual-Glo^®^ Luciferase Assay System (Promega) on an FLx800^TM^ fluorescence microplate reader (Biotek, Winooski, VT, USA). The relative firefly luciferase activity was normalized against the renilla luciferase activity.

The tannic acid was dissolved in a small aliquot of acetone and then serially diluted in acetone to test concentrations of 0.01, 0.1 and 1 mg/mL. Sf9 cells were seeded onto a 96-well plate (9 × 10^4^ cells/well) and transiently cotransfected with *MtGST* promoter-*PGL 4.10* luciferase reporter constructs (200 ng/well) and the internal *renilla* luciferase reporter plasmid phRL-TK (Promega; 20 ng/well) using Cellfectin II reagent (Invitrogen; 1 μL/well). Five hours post-transfection, tannic acid or equal volumes of acetone were added to the wells. After 48 h, the cells were harvested, and the resulting lysates were used to measure the renilla and firefly luciferase activities with a Dual-Glo^®^ Luciferase Assay System (Promega) on an FLx800^TM^ fluorescence microplate reader (Biotek, Winooski, VT, USA). The relative firefly luciferase activity was normalized against the renilla luciferase activity reported for each construct. The induction folds reported are expressed as a ratio of the normalized tannic acid-induced firefly luciferase activity to the normalized basal firefly luciferase activity (acetone control).

### 2.9. Data Analysis

Data collected from these assays were subjected to analysis of variance using InStat software (GraphPad, San Diego, CA, USA). A Student’s *t*-test followed by a two-tailed unpaired *t*-test was used to compare the significant differences of all two-sample. The statistical significance of multiple sample comparisons was calculated using a one-way analysis of variance followed by Tukey’s multiple comparisons. A value of *P <* 0.05 was considered statistically significant.

## 3. Results

### 3.1. Cloning and Identity of the GST cDNA

**Figure 1:**
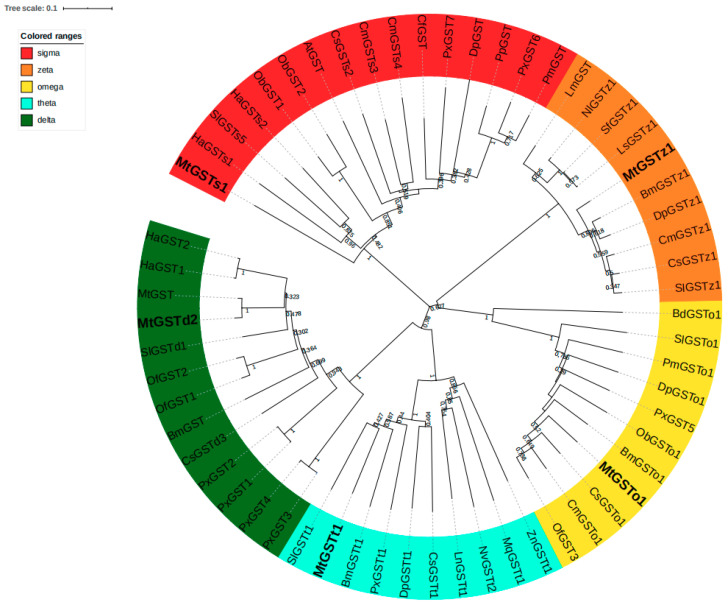
Phylogenetic relationships of 5 glutathione S-transferase (GST) genes of *M. troglodyta* with 55 GST proteins from 23 species.

Five different GST genes were identified in *M. troglodyta*. These GST cDNA sequences and their deduced amino acid sequences were deposited in GenBank with the following accession numbers: KU 963403, KU 963404, KU 963405, KU 963408 and KU 963410. The identities of these GST genes were revealed by a BLASTX search against the NCBI non-redundant database, and the optimal alignment of cloned sequences for Blastp is summarized in Supplementary Table S1. A phylogenetic analysis of the five GSTs deduced from their cDNA revealed that the GSTs belonged to five different cytosolic classes, delta (*MtGSTd2*), omega (*MtGSTo1*), sigma (*MtGSTs1*), theta (*MtGSTt1*), and zeta (*MtGSTz1*). The assignment of the five GSTs to the five classes was clearly supported by sequence similarity analysis. Fifty-five GST genes from 23 species, such as *Spodoptera litura*, *Papilio xuthus*, *Bombyx mori* and *Cnaphalocrocis medinalis*, showed different degrees of genetic relationships with the five GST genes. The percentages of identity of the deduced amino acids for the five GSTs were 43%–95% relative to other 55 GST genes established in the phylogenetic tree, and MtGSTd2 showed the strongest genetic relationship with GSTd of *Bombyx mori* and *Spodoptera litura*. MtGSTo1 was closely related to CsGSTo1, MtGSTs1 shared the highest identity with CfGST, and MtGSTt1 and MtGSTz1 shared the highest identity with SlGSTt1 and SlGSTz1, respectively (Figure 1).

The phylogenetic tree was constructed using the neighbor joining method (NJ), and the P-distances modeling and a pairwise deletion of gaps were performed by the MEGA 6.0 software package. The reliability of the tree structure and node support was evaluated by bootstrap analysis with 1000 replicates. The GenBank accession numbers of the sequences used in this tree are listed in Table S2.

At: *Amyelois transitella*; Bd: *Bactrocera dorsalis*; Bm: *Bombyx mori*; Cf: *Choristoneura fumiferana*; Cm: *Cnaphalocrocis medinalis*; Cs: *Chilo suppressalis*; Dp: *Danaus plexippus*; Ha: *Helicoverpa armigera*; Lm: *Locusta migratoria*; Ln: *Lasius niger*; Ls: *Laodelphax striatella*; Mq: *Melipona quadrifasciata*; Mt: *Micromelalopha troglodyta*; Nl: *Nilaparvata lugens*; Nv: *Nasonia vitripennis*; Ob: *Operophtera brumata*; Of: *Ostrinia furnacalis*; Pm: *Papilio machaon*; Pp: *Papilio polytes*; Px: *Papilio xuthus*; Sf: *Sogatella furcifera*; Sl: *Spodoptera litura*; Zn: *Zootermopsis nevadensis*.

### 3.2. Induction of GST Gene Expression in M. troglodyta by Tannic Acid

**Figure 2:**
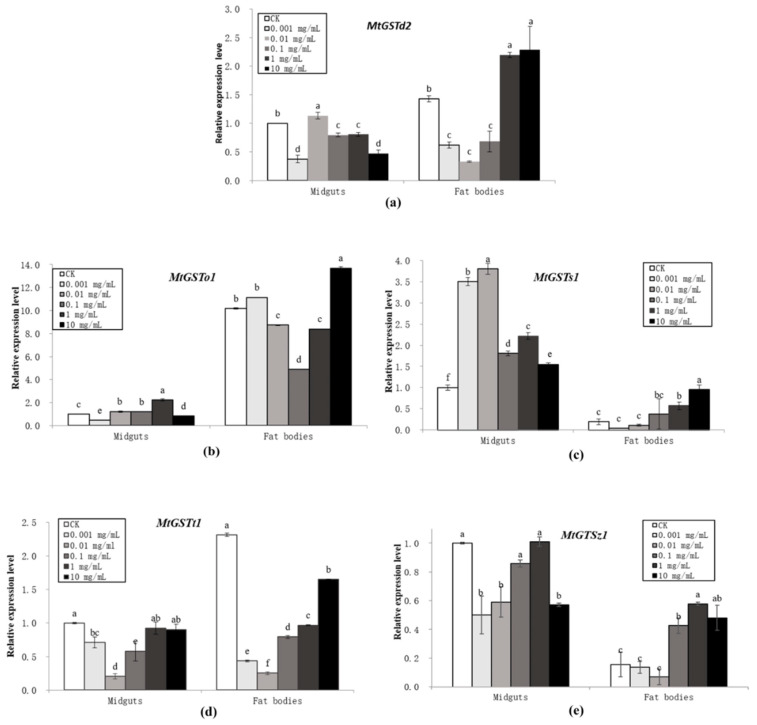
Quantification of relative expression of the five GST genes in *M. troglodyta* exposed to double-distilled water as a control (0) and to tannic acid at five different concentrations (0.001, 0.01, 0.1, 1, and 10 mg/mL) at 96 h posttreatment (**a**) *MtGSTd2*; (**b**) *MtGSTo1*; (**c**) *MtGSTs1*; (**d**) *MtGSTt1*; (**e**) *MtGSTz1*.

The expression of the five GST mRNAs in the midguts and fat bodies of *M. troglodyta* larvae was clearly induced by exposure to five different concentrations of tannic acid, 0.001, 0.01, 0.1, 1, and 10 mg/mL, for 96 h (Figure 2). The effects of tannic acid on the mRNA expression of GST genes at 96 h were compared with those of the double-distilled water treatment (control) and the treatments with different concentrations of tannic acid at 96 h post-treatment (Figure 2). A statistical analysis of the expression results showed that some differences were significant. For *MtGSTd2*, 0.01 mg/mL tannic acid increased while other treatments reduced the expression in midguts (F = 164.30; df = 5,18; *P* < 0.0001), and 1 and 10 mg/mL tannic acid increased while other treatments reduced the expression in fat bodies (F = 81.07; df = 5,18; *P* < 0.0001). The mRNA expression of *MtGSTo1* was significantly upregulated at a concentration of 0.01–1 mg/mL, whereas other concentrations of tannic acid caused significant downregulation in midguts (F = 448.22; df = 5,18; *P* < 0.0001); the mRNA expression of *MtGSTo1* was significantly upregulated at a concentration of 10 mg/mL, whereas 0.01-1mg/mL tannic acid caused significant downregulation in fat bodies (F = 10192; df = 5,18; *P* < 0.0001). All five concentrations of tannic acid significantly induced the expression of *MtGSTs1* in midguts (F = 745.80; df = 5,18; *P* < 0.0001), and 1 and 10 mg/mL tannic acid induced the expression of *MtGSTs1* mRNA in the fat bodies (F = 19.153; df = 5,18; *P* < 0.0001). Tannic acid caused significant downregulation of *MtGSTt1* mRNA expression at concentrations of 0.001–0.1 mg/mL (F = 47.67; df = 5,18; *P* < 0.0001), and all five concentrations of tannic acid decreased *MtGSTt1* mRNA expression in fat bodies (F = 8350.8; df = 5,18; *P* < 0.0001). The expression of *MtGSTz1* mRNA was significant downregulated by 0.001, 0.01 and 10 mg/mL tannic acid in midguts (F = 41.30; df = 5,18; *P* < 0.0001); the expression of *MtGSTz1* mRNA was significantly upregulated by 0.1–10 mg/mL tannic acid (F = 48.32; df = 5,18; *P* < 0.0001) (Figure 2).

The mRNA levels in the control and each treatment were normalized using to the mRNA level of actin, the reference gene. The mean expression in each treatment was shown as the fold change from the mean expression in the control. The vertical bars indicate standard deviations of the mean (n = 4). The statistical significance of the gene expressions was calculated using a one-way analysis of variance followed by Tukey’s multiple comparisons; a Student’s *t*-test followed by a two-tailed unpaired *t*-test was used to compare the significant differences between midguts and fat bodies. A value of *P* < 0.05 was considered statistically significant.

In this study, tissue-specific expression patterns of the genes were analysed. All five GST genes had different expression levels between midguts and fat bodies. *MtGSTd2* (t = 15.93; df = 6; *P* < 0.0001)*, MtGSTo1* (t = 298.27; df = 6; *P* < 0.0001) and *MtGSTt1* (t = 97.30; df = 6; *P* < 0.0001) showed higher expression levels in fat bodies; however, *MtGSTs1* (t = 17.63; df = 6; *P* < 0.0001) and *MtGSTz1* (t = 19.63; df = 6; *P* < 0.0001) had higher expression in midguts. For instance, the expression of *MtGSTo1* in fat bodies was 10 times higher than that in midguts, and *MtGSTz1* expression was more than 6 times higher in midguts than in fat bodies (Figure 2).

### 3.3. Characterization of the 5′-Flanking Promoter Sequences of the Five MtGST Genes

Sequences of the upstream portion of the 5 *MtGST*s were obtained by genome walking methods (Figures S1–S5). Sequence analysis showed that the terminal sequences of these clones were identical to the *MtGST* cDNA sequences, which confirmed that the correct fragments of the upstream sequences of the five *MtGST*s had been amplified. Software was used to analyse the upstream sequences and predict the locations of the transcription start sites. The nucleotides were numbered relative to the transcription start sites (TSSs) indicated by +1, with upstream sequences preceded by a ‘−’ and downstream sequences preceded by a ‘+’. Based on sequence analysis, the typical characteristics were predicted, such as the recognition site CCAAT/enhancer-binding protein alpha (C/EBPalp).

### 3.4. Functional Analysis of MtGST Promoters

**Figure 3:**
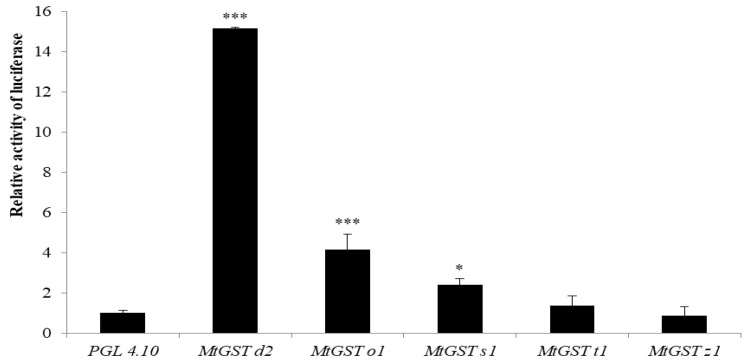
The promoter activity was measured as luciferase activity in Sf9 cells. * on the bars indicate that the means are significantly different between the control and *MtGST* promoters (*** means *P* < 0.001, * means *P* < 0.05).

The restructured plasmids were transferred into Sf9 cells by lipofectin-mediated transfection and luciferase assays were performed. At 48 h after transfection, *MtGSTd2* showed the highest promoter strength, which was more than 15 times that of the control. *MtGSTo1* and *MtGSTs1* exhibited promoter activity, albeit at weaker levels than *MtGSTd2*, but still showed strong promoter activity in Sf9 cells. There was no significant difference between *MtGSTt1*, *MtGSTz1* and the control promoter strengths (Figure 3).

The relative luciferase activity of PGL 4.10 was defined as 1, and the PGL 4.10 promoter was used as a positive control. The vertical bars indicate standard deviations of the mean (n = 4).

**Figure 4:**
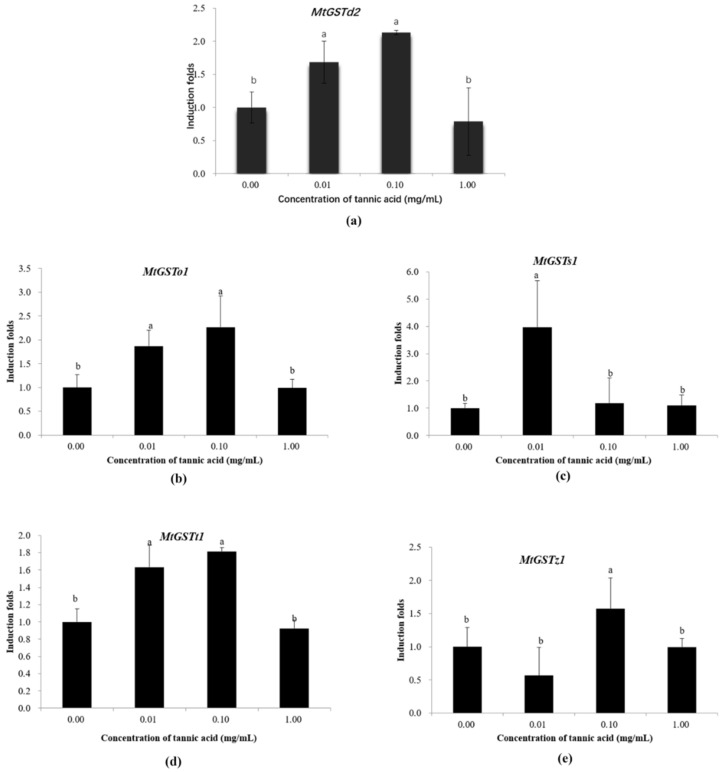
The effect of tannic acid on promoter activities. (**a**) *MtGSTd2*; (**b**) *MtGSTo1*; (**c**) *MtGSTs1*; (**d**) *MtGSTt1*; (**e**) *MtGSTz1*.

The restructured plasmids were transferred into Sf9 cells by lipofectin-mediated transfection, and luciferase assays were performed in the presence of tannic acid. At 48 h after transfection, the *MtGSTd2* (F = 14.49; df = 3,12; *P* = 0.0003), *MtGSTo1* (F = 10.09; df = 3,12; *P* = 0.0013) and *MtGSTt1* (F = 33.48; df = 3,12; *P* < 0.0001) promoter activities were induced by 0.01 and 0.1 mg/mL tannic acid, and the *MtGSTs1* (F = 8.66; df = 3,12; *P* = 0.0025) and *MtGSTz1* (F = 5.56; df = 3,12; *P* = 0.0126) promoter activities were increased by 0.01 or 0.1 mg/mL tannic acid, respectively (Figure 4).

Promoter activities were measured as the luciferase activities in Sf9 cells. The normalized basal firefly luciferase activities (acetone control) were defined as 1, and the normalized basal firefly luciferase activities (acetone control) were used as a positive control. The vertical bars indicate standard deviations of the mean (n = 4). The statistical significance of the induction folds was calculated using a one-way analysis of variance followed by Tukey’s multiple comparisons, a value of *P* < 0.05 was considered statistically significant.

## 4. Discussion

Based on their subcellular location, GSTs were classified into three categories: cytosolic GSTs, microsomal GSTs and mitochondrial GSTs [29]. Insect cytosolic GSTs were further divided into omega, sigma, epsilon, delta, thera and zeta classes based on amino acid sequences [30,31,32]. The five GSTs cloned from *M. troglodyta* in this study belonged to the omega, sigma, delta, thera and zeta classes. With the development of molecular biology techniques, more GST genes have been cloned and identified in insects [33,34,35], for example, the GST from the spruce budworm *Choristoneura fumiferana* (Clem.) [36,37] and the delta class GST from *Drosophila melanogaster* [29]. The acquisition of these GST genes laid the foundation for the study of GST characteristics and the regulation of GST expression in insects.

In this paper, five GST genes, *MtGSTd2*, *MtGSTo1*, *MtGSTs1*, *MtGSTt1* and *MtGSTz1*, were cloned in *M. troglodyta*. Although we reported that *MtGSTd1* mRNA expression was induced by tannic acid [19], the induction of GST activity by tannic acid was the overall result for all GST gene expression in vivo. Therefore, our results showed that the mRNA expression of four GST genes, *MtGSTd2*, *MtGSTo1*, *MtGSTs1* and *MtGSTz1*, was also induced by tannic acid. These results were consistent with those of other studies. For example, Feng et al. reported that balsam fir foliage induced the expression of CfGST mRNA in *Choristoneura fumiferana* [36], and gstD1 and gstD21 mRNA expression in *D. melanogaster* was induced by phenobarbita [38]. These results showed that the increase in GST activities was mainly due to changes at the transcriptional level.

Plant secondary metabolites are inducers of insect GSTs in vivo. An increase in aphid GST activity was found in response to phenolic acids [39]; the GST activities of midguts were significantly induced by geranium petals and quisqualic acid in *Popillia japonica* Newman [40]; the GST activities were induced by quercetin in the silkworm [41]. This induction process appears to be an adaptation mechanism of organisms to counter chemical stress. Tannic acid is characterized as a plant polyphenol and is commonly distributed in many plants. Our previous results suggested that GST activities in insects were induced by feeding upon or exposure to tannic acid [19], which was in agreement with the response of insect herbivores to plant allelochemicals including the induction of GST activity by tannic acid in *H. armigera* [23]. We found that the increase in GST activity was mainly due to the increased expression of GST mRNA. However, little information is available on the molecular mechanisms by which GST mRNA expression is induced by tannic acid in insects. The isolation and characterization of promoters is helpful for the study of gene expression and regulation and provides basic materials for functional studies. However, there are no reports on GST promoters in *M. troglodyta* at home and abroad. Therefore, we studied the GST promoters of *M. troglodyta* for the first time. In this paper, the five promoter sequences of the five *MtGST* genes were cloned, which was very important in clarifying the molecular mechanisms of GST expression and regulation in *M. troglodyta*.

Plant secondary metabolites have an inhibitory effect on GST activities in vitro. For examples, Tang et al. showed that tannic acid inhibited GST activities in *M. troglodyta* and *Clostera anachoreta* (Fabricius) [21]; Tang et al. reported that GST activities from *Odontotermes formosanus* (Shiraki) and *Reticulitermes chinensis* Snyder were inhibited by tannic acid [42]. In addition, the rat liver GST was inhibited by plant polyphenols [20]; GSTs were inhibited by allelochemicals in cotton bollworm, *H. armigera* [43]; GSTs were inhibited by allelochemicals in the fall armyworm, *Spodoptera frugiperda* [44]. However, in this experiment, the restructured plasmids were transferred into Sf9 cells by lipofectin-mediated transfection, and luciferase assays were performed in the presence of tannic acid. The results showed that the GST promoter activities were induced by tannic acid. The difference may be due to one in vitro reaction and one in vivo reaction. In in vitro inhibition of GST activities, plant secondary metabolites react directly with GST protein; in in vivo induction of GST promoter activities, tannic acid need to enter Sf9 cells to react with GST promoters.

Currently, promoters are a research hotspot in genetic studies as some of the most important *cis*-acting elements in gene expression and regulation. Many inducible promoters have been identified in attempting to understand the molecular mechanisms of expression and regulation. A stress-induced gene (ZmRXO1) promoter was cloned and analysed in maize [45]. Fujita et al. found that the *BmNPV ie-1* promoter was involved in gene expression in various organisms, including insects, mammals, plants and bacteria [46]. Moreover, many functional elements and coregulatory binding sites for the nucleus were discovered. For example, kB and GATA factors alone were not sufficient to activate moricin expression in *Manduca sexta*, and the kB-GATA element from the *Ms* moricin promoter could significantly increase the activities of *Drosophila melanogaster* AMP gene promoters. Furthermore, a moricin promoter activating element (MPAE), which may contain coregulatory binding sites for nuclear factors specifically expressed in lepidopteran species, could increase the activity of the *drosomycin* promoter [47]. The transcription factor Sp1 was shown to promote human *LRRK2* gene promoter activity and gene expression, whereas its inhibitor MTM reduced promoter activity and gene expression [48]. However, little information is available on the GST promoter in insects. Chen et al. showed that the *Slgste1* promoter played an important role in regulating gene expression in response to phytochemicals and insecticides in *Spodoptera litura* [17]; Hu et al. reported that in the promoter region of the *SeGST* gene from *Spodoptera exigua*, *cis*-acting elements responded to chlorpyrifos and cypermethrin [18]. However, there have been no previous reports on the GST promoter in Notodontidae insects. In this paper, five GST gene promoters were isolated, and the activities of these five promoters under tannic acid stress were studied in *M. troglodyta*. The functional analysis results indicated that the promoter activities of *MtGSTd2, MtGSTo1*, *MtGSTz1* and *MtGSTs1* were induced by tannic acid, which was consistent with the transcriptional expression of these genes. These results showed that tannic acid could influence the activities of the GST promoters, and the GST promoters further regulated the transcriptional expression of GST genes in *M. troglodyta*. In addition, the promoter activity of *MtGSTt1* was induced by tannic acid, while the transcriptional expression of *MtGSTt1* was inhibited, which showed that in addition to the promoter, there may be negative regulatory elements involved in the expression of *MtGSTt1* in *M. troglodyta*. Therefore, in future studies, we will focus on the core elements and response elements to verify the function of *MtGST* promoters and determine the regulatory mechanism controlling expression.

## 5. Conclusions

In summary, five GST genes were identified from *M. troglodyta*. These five GST genes were classified into five different cytosolic GST classes: delta, omega, sigma, theta, and zeta. The present study provided an overview of the five GST expression profiles under tannic acid stress in insect midguts and fat bodies. Moreover, the five *MtGST* promoters were isolated and analysed, which was firstly obtained in Notodontidae insects. Furthermore, the functional analysis results indicated that all four *MtGST* promoter activities, i.e., those of *MtGSTd2, MtGSTo1*, *MtGSTz1* and *MtGSTs1*, were induced by tannic acid, which was consistent with the transcriptional expression of these genes, showing that the effects of tannic acid on the mRNA expression of GST genes as a result of promoter activity varied. However, the promoter activity of *MtGSTt1* was induced by tannic acid, while the transcriptional expression of *MtGSTt1* was inhibited, which showed that in addition to the promoter, there may be negative regulatory elements involved in the expression of *MtGSTt1* in *M. troglodyta*. These results provide an important theoretical basis for elucidating the mechanism regulating tannic acid effects on the expression of GSTs. This will aid in deepening the understanding of the interactions between GST and tannic acid in *M. troglodyta* and is highly significant for the comprehensive management of this pest.

## Figures and Tables

**Table 1:** Primers used in experiments.

GenBank	Gene	Primer	Sequence (5′-3′)	Annealing Temperatures (°C)	Amplicon Size (bp)	Application
KU 963403	*MtGSTd2*	F	TCAGCGTTTGAAGATGTCG	53	678	ORF
		R	GCTTCTTACAGCTCGGTTTTAG			ORF
		Q-F	AAAGCCGATGAAGCCAAGTT	60	105	qPCR
		Q-R	TGCCAGGGTCAGTTTATCTCC			qPCR
		Common 1	GTAATACGACTCACTATAGGGC	67	-	Genome Walker
		1	ATCGTCTACTATCGTGGGAATGGTGTG			Genome Walker
		Common 2	ACTATAGGGCACGCGTGGT	67	975	Genome Walker
		2	GACGAGCTTCAGGTTGAGTTGGATGT			Genome Walker
		F-1	AACTCGAGAGATTACTATAGGGCACG	62	920	Constructs
		R-1	AAGCTAGCAGGTAGTACAGGTCGATC			Constructs
KU 963404	*MtGSTo1*	F	TCGCTGCCATCATGTCTG	53	778	ORF
		R	GTTTATTCCTTCTTCTTCCTGG			ORF
		Q-F	ACTATACAGCTGCCTTCAACGC	60	153	qPCR
		Q-R	GCCACAATGTGAAGTCAACGAG			qPCR
		Common 1	GTAATACGACTCACTATAGGGC	67	-	Genome Walker
		1	ACTAAAACTGTTCTCTCGGCGTATGGG			Genome Walker
		Common 2	ACTATAGGGCACGCGTGGT	67	977	Genome Walker
		2	GGGCAGAATCTCATAGCGAATACACG			Genome Walker
		F-1	AACTCGAGATTACTATAGGGCACGC	62	847	Constructs
		R-1	AAGCTAGCGGTTTGTAAATGTTTTTC			Constructs
KU 963405	*MtGSTs1*	F	GAGTCCTTGACAATGGCTA	53	660	ORF
		R	GCTCGCTATTGCACAACC			ORF
		Q-F	GACTTTTGGGCCAACATCAG	60	112	qPCR
		Q-R	CAAATCTGGGCAAGAAGAACAC			qPCR
		Common 1	GTAATACGACTCACTATAGGGC	67	-	Genome Walker
		1	AACCTTACATCCTCAAATTCCTGTTTAGTG			Genome Walker
		Common 2	ACTATAGGGCACGCGTGGT	67	1012	Genome Walker
		2	GCTAAGGCAGGTGCTTCAAAATAATACAG			Genome Walker
		F-1	AACTCGAGCGACGAAGGCTT	62	926	Constructs
		R-1	AAGCTAGCATTTGCTGCACTATCA			Constructs
KU 963408	*MtGSTt1*	F	GTTCAATACCTTCAAGTTTTTC	53	722	ORF
		R	ACACTTTAGACTTAACTTTAGACTGC			ORF
		Q-F	CCACTGTCGCTGATCTGCTG	60	126	qPCR
		Q-R	AGGGGCTGAAATGTCGTTG			qPCR
		Common 1	GTAATACGACTCACTATAGGGC	67	-	Genome Walker
		1	CAAAGTACAAGAACATAGCCGAGTGGTG			Genome Walker
		Common 2	ACTATAGGGCACGCGTGGT	67	1894	Genome Walker
		2	TCTTCACAGAGTGGTCGTTATTTCAGTTC			Genome Walker
		F-1	AACTCGAGTGCCTGCAGGTC	62	1853	Constructs
		R-1	AAGCTAGCGCTTTGATTTGGTC			Constructs
KU 963410	*MtGSTz1*	F	CTCAAAATACAACGGGAACC	53	707	ORF
		R	CGACCGTGACAAGAGGC			ORF
		Q-F	AGTCAATCCGATGGAGCAGG	60	180	qPCR
		Q-R	GGTTGGATGCCTGATGCTATT			qPCR
		Common 1	GTAATACGACTCACTATAGGGC	67	-	Genome Walker
		1	CTTGTCTCTTCCAGGTAGTGCATTATGTTC			Genome Walker
		Common 2	ACTATAGGGCACGCGTGGT	67	1590	Genome Walker
		2	ATGGGATCTCCTTCAAGTTGAGTGCG			Genome Walker
		F-1	AACTCGAGGGCACGCGTG	62	1177	Constructs
		R-1	AAGCTAGCTTGTAAGTCGGTATGTATGTAA			Constructs
GU 262991	*Actin*	Q-F	CTCTGGTCGACTTGAGGCTGGAC	60	241	qPCR
		Q-R	CTCTGGTCGACTTGAGGCTGGAC			qPCR

Primers were designed using Premier 5.0 software and synthesized by Shanghai Generay Biotechnology Co., Ltd. F, forward; R, reverse. Enzyme cutting sites are underlined.

## Supplementary Materials

The following materials are available online at https://www.mdpi.com/2075-4450/11/6/339/s1: Figure S1: *MtGSTd2* promoter and part of the 5′ end of the coding sequence, Figure S2: *MtGSTo1* promoter and part of the 5′ end of the coding sequence, Figure S3: *MtGSTs1* promoter and part of the 5′ end of the coding sequence, Figure S4: *MtGSTt1* promoter and part of the 5′ end of the coding sequence, Figure S5: *MtGSTz1* promoter and part of the 5′ end of the coding sequence, Table S1: The optimal alignment of cloned sequences for Blastp, Table S2: The accession numbers for the other sequences included in the phylogenetic analyses.
