# Supplementary material for: Molecular Cloning and Characterization of Five Glutathione S-Transferase Genes and Promoters from Micromelalopha troglodyta (Graeser) (Lepidoptera: Notodontidae) and Their Response to Tannic Acid Stress
Source: Insects. 2020 Jun 1;11(6):339. doi: 10.3390/insects11060339 (PMC7349759; doi:10.3390/insects11060339)
Supplement: Supplementary file 1 [file insects-11-00339-s001.zip › supplementary files/Table S1.docx]

**Table S1** The optimal alignment of cloned sequences for Blastp

|  | Best Blatsp Mach | | | |
| --- | --- | --- | --- | --- |
| Gene | Species | Accession number | E-value | Identities |
| MtGSTd2 | *Manduca sexta* | XP_030035463.1 | 2e-122 | 78% |
| MtGSTz1 | *Cydia pomonella* | ARM39005.1 | 9e-151 | 95% |
| MtGSTt1 | *Amyelois transitella* | XP_013194289.1 | 4e-120 | 74% |
| MtGSTs1 | *Trichoplusia ni* | XP_026732226.1 | 1e-79 | 60% |
| MtGSTo1 | *Chilo suppressalis* | AKS40345.1 | 4e-164 | 87% |
