# Supplementary material for: Molecular Cloning and Characterization of Five Glutathione S-Transferase Genes and Promoters from Micromelalopha troglodyta (Graeser) (Lepidoptera: Notodontidae) and Their Response to Tannic Acid Stress
Source: Insects. 2020 Jun 1;11(6):339. doi: 10.3390/insects11060339 (PMC7349759; doi:10.3390/insects11060339)
Supplement: Supplementary file 1 [file insects-11-00339-s001.zip › supplementary files/Table S2.docx]

**Table S2** The accession numbers for the other sequences included in the phylogenetic analyses

| **Gene** | **Accession number** |
| --- | --- |
| HaGST1 | ADD17089.1 |
| HaGST2 | ABK40535.1 |
| PxGST1 | BAM18511.1 |
| BmGST | NP_001037546.1 |
| MtGST | ACT98684.1 |
| OfGST1 | AEF98444.1 |
| OfGST2 | AHV85207.1 |
| PxGST2 | KPJ04368.1 |
| CsGSTd3 | AKS40340.1 |
| PxGST3 | KPJ04369.1 |
| PxGST4 | KPJ20605.1 |
| SlGSTd1 | AIH07594.1 |
| CsGSTo1 | AKS40345.1 |
| CmGSTo1 | AIL29316.1 |
| PxGST5 | AHW45906.1 |
| BmGSTo1 | NP_001040131.1 |
| ObGSTo1 | KOB75341.1 |
| DpGSTo1 | EHJ65985.1 |
| PmGSTo1 | KPJ07248.1 |
| SlGSTo1 | AEG75845.1 |
| OfGST3 | AHV85208.1 |
| BdGSTo1 | AFJ05097.1 |
| CfGST | AAF23078.1 |
| DpGST | EHJ75427.1 |
| SlGSTs5 | AIH07592.1 |
| CmGSTs3 | AIL29320.1 |
| PpGST | XP_013142293.1 |
| PxGST6 | NP_001299208.1 |
| PxGST7 | NP_001296061.1 |
| CsGSTs2 | AKS40349.1 |
| AtGST | XP_013188171.1 |
| CmGSTs4 | AIZ46904.1 |
| HaGSTs1 | AIB07718.1 |
| HaGSTs2 | AIB07719.1 |
| ObGST1 | KOB62848.1 |
| ObGST2 | KOB75653.1 |
| PmGST | XP_014354690.1 |
| SlGSTt1 | AIH07600.1 |
| BmGSTt1 | NP_001108463.1 |
| CsGSTt1 | AKS40350.1 |
| DpGSTt1 | EHJ70012.1 |
| PxGSTt1 | KPI95695.1 |
| ZnGSTt1 | KDR15740.1 |
| NvGSTt2 | NP_001165925.1 |
| LnGSTt1 | KMQ97840.1 |
| MqGSTt1 | KOX71611.1 |
| CsGSTz1 | AKS40351.1 |
| BmGSTz1 | NP_001037418.1 |
| SlGSTz1 | AIH07598.1 |
| CmGSTz1 | AIL29321.1 |
| DpGSTz1 | EHJ73846.1 |
| LmGST | AHC08063.1 |
| SfGSTz1 | AFJ75819.1 |
| LsGSTz1 | AEY80030.1 |
| NlGSTz1 | AFJ75820.1 |
