# Supplementary figures and images for: Molecular Cloning and Characterization of Five Glutathione S-Transferase Genes and Promoters from Micromelalopha troglodyta (Graeser) (Lepidoptera: Notodontidae) and Their Response to Tannic Acid Stress
Source: Insects. 2020 Jun 1;11(6):339. doi: 10.3390/insects11060339 (PMC7349759; doi:10.3390/insects11060339)

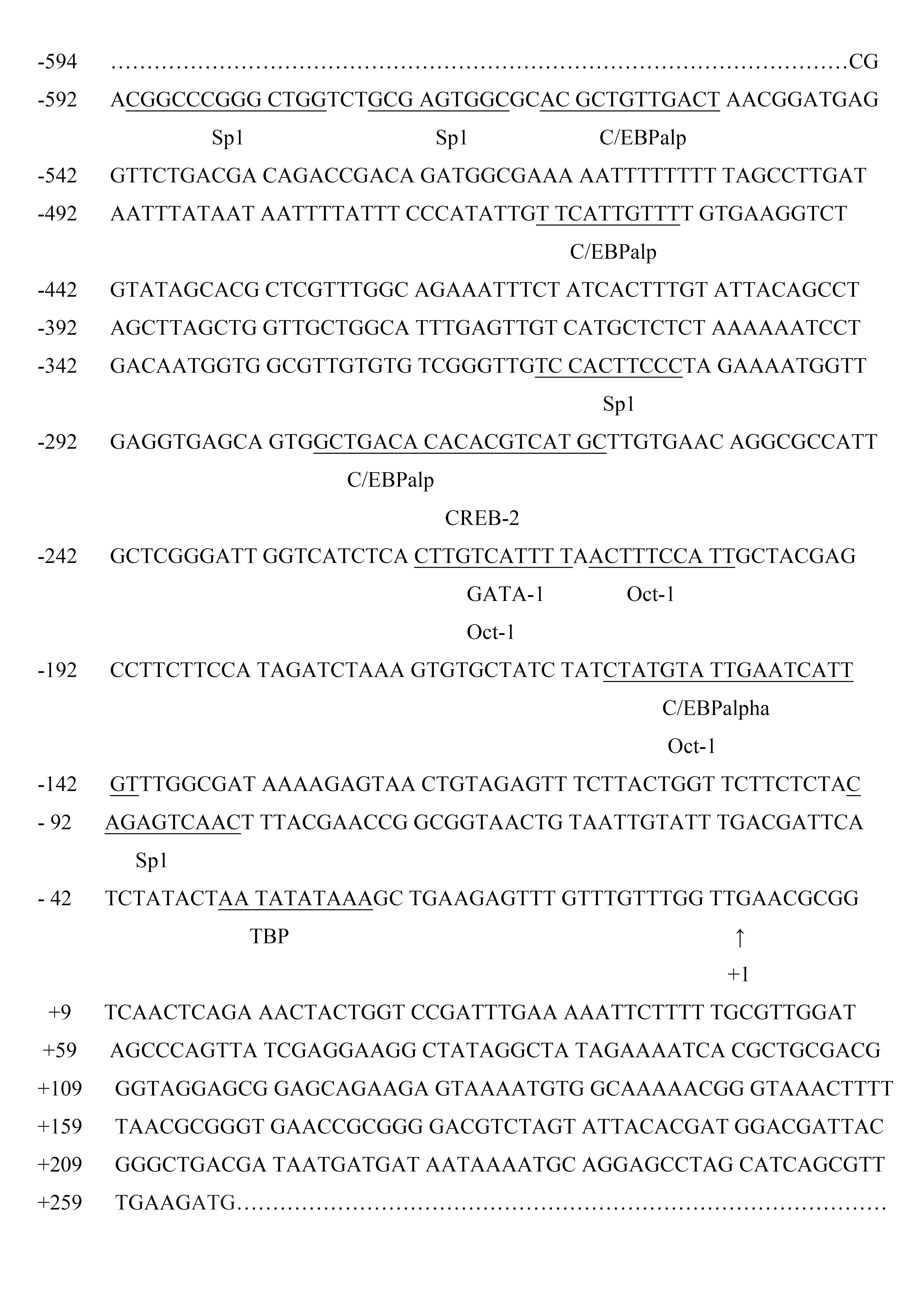

Supplement: Supplementary file 1 [file insects-11-00339-s001.zip › supplementary files/Figure S1 MtGSTd2.tif]

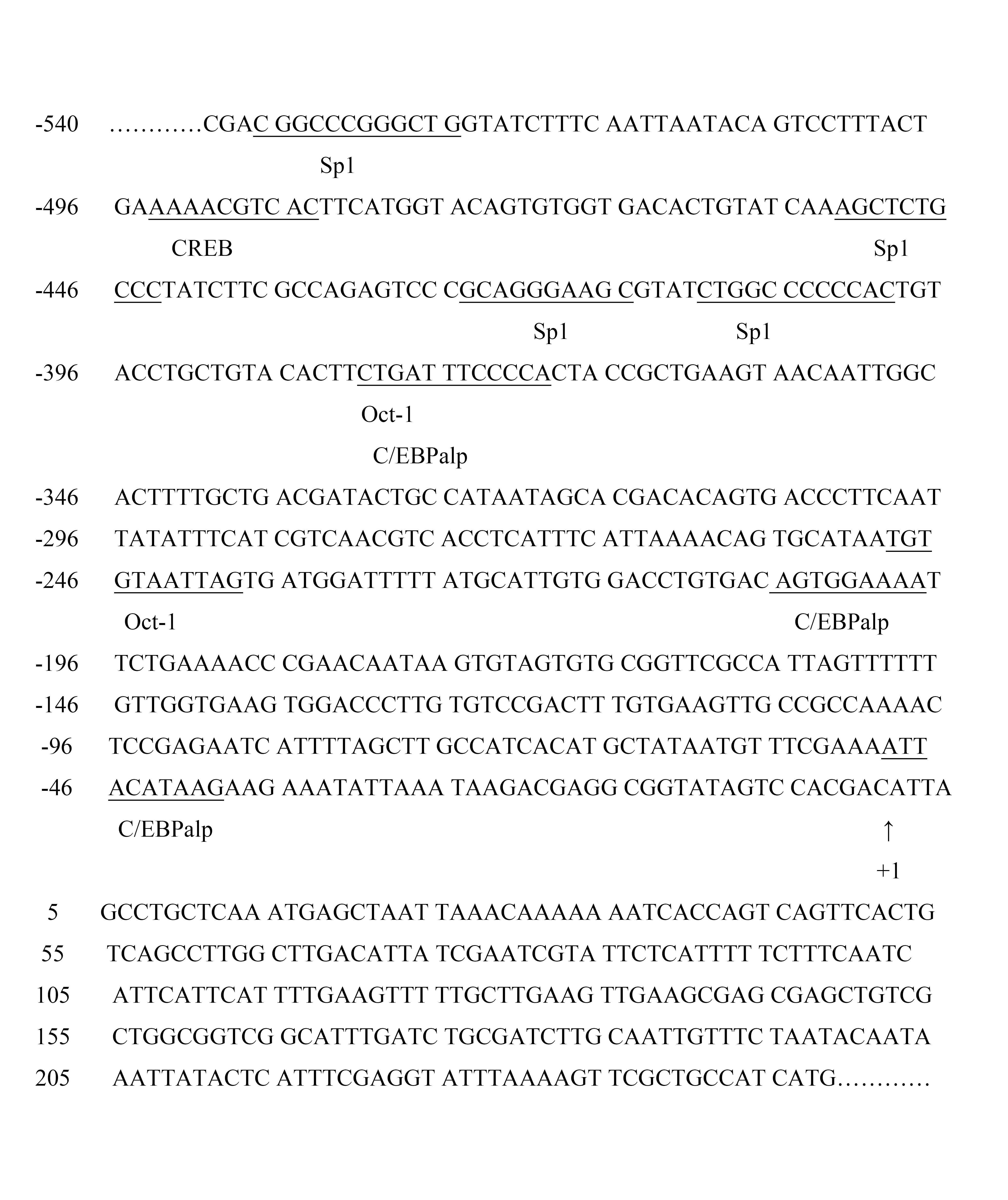

Supplement: Supplementary file 1 [file insects-11-00339-s001.zip › supplementary files/Figure S2 MtGSTo1.tif]

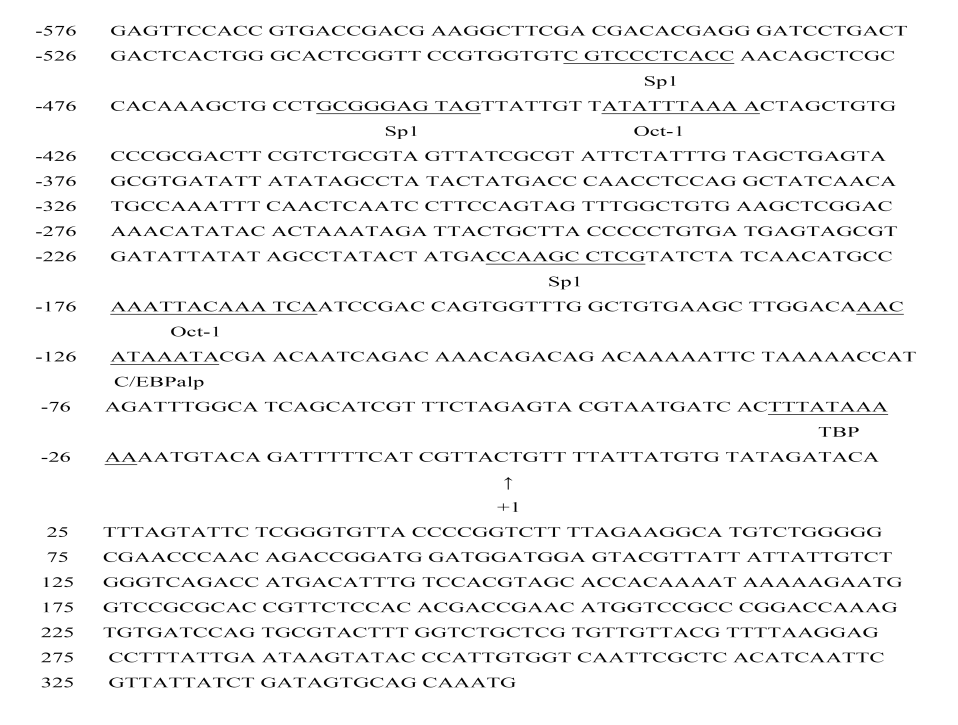

Supplement: Supplementary file 1 [file insects-11-00339-s001.zip › supplementary files/Figure S3 MtGSTs1.png]

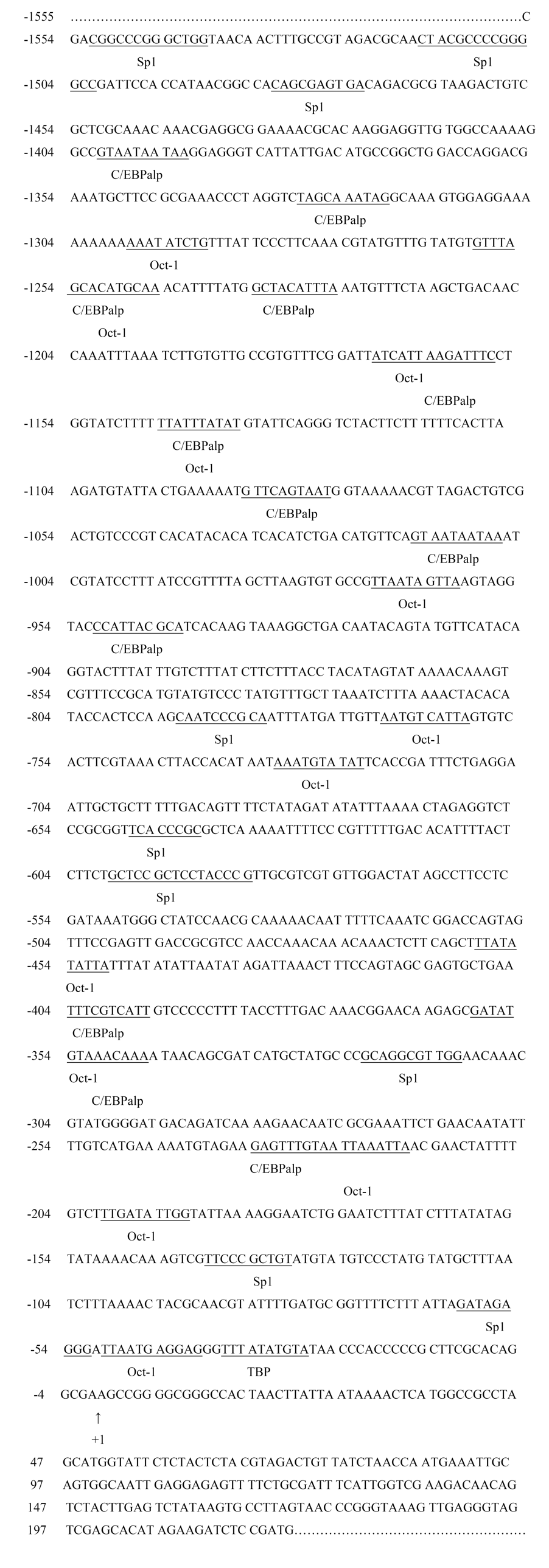

Supplement: Supplementary file 1 [file insects-11-00339-s001.zip › supplementary files/Figure S4 MtGSTt1.tif]

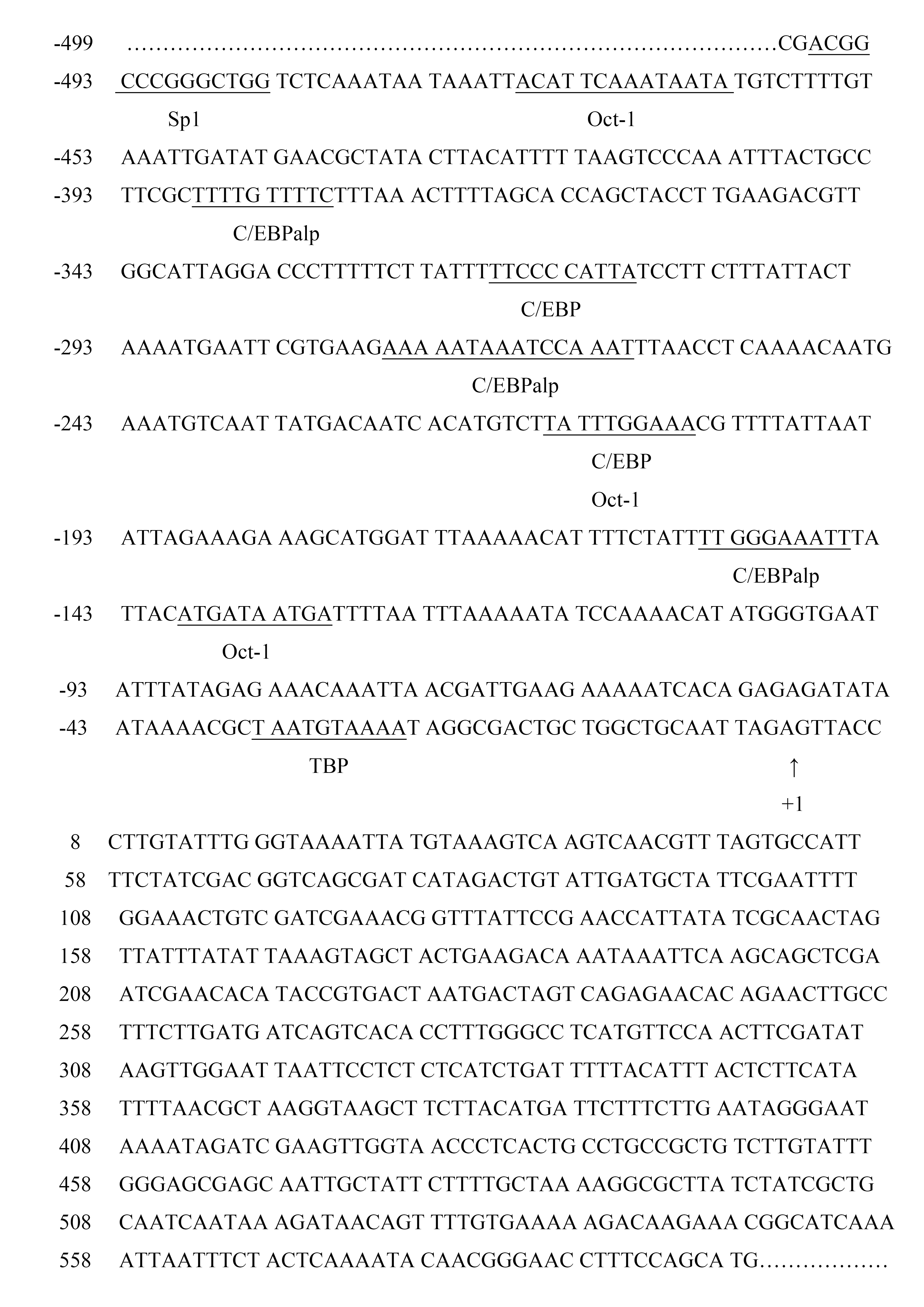

Supplement: Supplementary file 1 [file insects-11-00339-s001.zip › supplementary files/Figure S5 MtGSTz1.tif]
